# Assessment of Hand-Washing Knowledge and Practice among Nursing Undergraduates in Saudi Arabia

**DOI:** 10.1155/2024/7479845

**Published:** 2024-03-19

**Authors:** Wajid Syed, Mahmood Basil A. Al-Rawi

**Affiliations:** ^1^Department of Clinical Pharmacy, College of Pharmacy, King Saud University, Riyadh 11451, Saudi Arabia; ^2^Department of Optometry, College of Applied Medical Sciences, King Saud University, Riyadh 11451, Saudi Arabia

## Abstract

**Purpose:**

The present study investigated the knowledge and practice of hand hygiene among entry-level nursing students at King Saud University in Riyadh, Saudi Arabia. *Participants and Methods*: A cross-sectional, institutional-based study was conducted among Saudi nursing students over a period of four months at the King Saud University College of Nursing, using a structured, self-administered questionnaire that assessed demographics, knowledge, and the practice of hand washing.

**Results:**

A response rate of 95% was obtained. Of the 304 respondents, 66.1% (*n* = 201) were males. The majority of the students (94.2%) agreed that direct or indirect contact was the most important route for the transmission of healthcare-associated infection. In addition, most of the students washed their hands after contact with body secretions (89.5%), while 83.2% of them washed their hands before and after contact with patients. Furthermore, 83.6% of them applied soap water during hand washing, while 59.9% of the students used an alcohol-based hand rub for hand hygiene. Our results found that female students reported higher mean knowledge score of 10.09 ± 1.27, compared to male students 9.63 ± 1.48, indicating a significant association between the mean knowledge score of hand washing and gender (*p*=0.004). Similarly, the mean practice score was higher among female students (5.00 ± 1.25), in comparison to male students 4.62 ± 1.46, with a significant association between gender and mean practice score (*p*=0.037).

**Conclusion:**

The findings of this study revealed that Saudi nursing students exhibit adequate knowledge and practice of hand washing. However, the current findings revealed variation in the knowledge and practice scores with respect to gender. The present results could support students and health care professionals in improving their hygienic practice, which may help to provide the optimum therapeutic benefits to patients.

## 1. Introduction

The practice of hand washing is among the highest priorities of healthcare professionals in preventing the transmission of healthcare-associated infections (HCAIs) [1–3]. It was evidenced that practicing hand washing before and after each healthcare procedure can, to a great extent, lower the possibility of transmitting HCAIs [4–6]. Recent studies in Saudi Arabia on HCAIs found an overall prevalence of 6.8% (114 of 1666) with the most common types of infections being pneumonia (27.2%), urinary tract infections (20.2%), and bloodstream infections (10.5%) [7], and intensive care unit patients had the highest prevalence of HCAIs [8]. It has been found that 30% of HCAIs can be prevented by primary measures, such as hand hygiene, and that hand washing is the most effective single behavior for stopping the spread of infection [9, 10].

In spite of the availability of extensive guidelines by the World Health Organization (WHO), adherence to hand washing among healthcare workers is low, rarely exceeding 50%, and thus represents a critical issue in healthcare settings [9, 10]. However higher compliance was reported among nurses [11–13] than amongst physicians [14]. Furthermore, the WHO developed five hand hygiene moments to reduce the risk of infection transmission among patients and health care workers [15]. Moment one is concerned with hand hygiene when entering the patient's zone before touching the patient, while moment two is concerned with hand hygiene immediately before the procedure, moment three is concerned with hand hygiene immediately after a procedure, moment four is concerned with hand hygiene after touching a patient, and moment five is concerned with hand hygiene after touching a patient's surroundings, even when the patient has not been touched [15].

Despite the fact that health care provider' hands might be a source of infection, environment where the healthcare providers' is working, another probable source of infection transmission [16]. For example, the use of computers and electronic instruments is increasing in all fields of healthcare, where many professionals may use the same keyboard [16]. Additionally, the fact that professionals eat at their workstation, where food crumbs remaining on the keyboard may form a medium favoring the growth of microbes [16, 17]. Furthermore, there were droplets of saliva that undoubtedly fell on the keyboard during talking, sneezing, and coughing, creating a probable vehicle for infection transmission, implying that all the surrounding things that we touch may serve as a vehicle for HCAIs [16]. Therefore, it is advisable to observe the general rules of hygiene and to clean hands before and after each healthcare procedure.

In Saudi Arabia, earlier data showed a significant difference between male and female nursing students in their knowledge and practice of hand washing [18]. Furthermore, a study conducted in 2016 reported a moderate level of hand-washing knowledge among Saudi nursing students [19], while studies performed in Singapore and Italy [1, 6] reported good hand washing practices among nursing students. Although it is well known that nursing students and practitioners had direct contact with patients during their workdays, clinical rotations, and internships [19]. Without proper hand washing, they could act as a vehicle for cross-contamination from the environment to the patients [19, 20]. In spite of this important practice, some studies have reported that nursing students had lower levels of knowledge about hand washing [18, 21]. The levels of knowledge and understanding concerning hand washing were associated with good compliance and practice [19]. Furthermore, earlier studies found that lack of experience, inadequate resources, lack of time, absence of disinfectants are the barriers for hand washing practice among health care professionals [5–8].

Healthcare infections are serious concern among the professionals and patients. The viruses and bacteria's behind the infections was sometimes transmitted through hands of physicians or nurses, to patients, and hand washing played a critical role in reducing its transmission [22]. Proper hand washing could guard against the droplet transmission of the most dangerous viruses, as well as other infectious disease viruses such as influenza. The WHO recommended frequent hand washing or the use of an alcohol-based hand rub, for both healthcare professionals and the public as an essential precaution to prevent the spread of the virus [22]. Therefore, it was essential to evaluate Saudi nursing students' knowledge and practice of hand washing to minimize the possibility of HCAIs. The objective of the present study was to assess the hand-washing knowledge and practice of entry-level nursing students at the King Saud University College of Nursing in Riyadh, Saudi Arabia.

## 2. Materials and Methods

### 2.1. Study Design and Settings

A cross-sectional, institutional-based study was conducted among nursing students at King Saud University College of Nursing in Riyadh, Saudi Arabia. The study was carried out from September to December 2019 for a total of four months. First year through third year entry-level nursing students was given a standardized, self-administered questionnaire. Students from other disciplines and those who did not speak Arabic were excluded. A senior researcher with significant survey design experience reviewed the questionnaire once it had been produced, following a thorough analysis of the existing literature on the subject [14, 18, 19]. The questionnaires were subjected to a translation procedure through forward and backward translation with the help of an Arabic speaker. In order to verify the accuracy of the questionnaire, the pilot study employed premade Arabic versions. Before distributing the questionnaire, a pilot test consisting of twenty students was undertaken to assess its readability and level of difficulty. The primary study excluded the pilot research's findings. The knowledge and perception surveys have Cronbach's alpha values of 0.81 and 0.79, respectively.

In this study, we defined healthcare-associated infection as those caused by viral, bacterial, or fungal pathogens and necessitated frequent hand washing and/or adherence to hand washing guidelines to prevent infection spread. While proper and consistent hand washing means washing hands with plain or antimicrobial soap and water after and before contact with the patients [23].

### 2.2. Study Questionnaires and Procedure

The questionnaire contained three sets [14]. The first set addressed demographic variables, including gender, educational level, and marital status, via multiple-choice questions. The second set consisted of hand-washing knowledge-related questions, with a total of 12 items evaluated via three choices (“yes”/“no”/“I don't know”). The last set of questions was related to the practice of hand hygiene and was based on a three-point Likert-type scale (Always, Sometimes, and Never). The completed anonymous questionnaires by the respondents were, checked for the accuracy and entered into the statistical package for social science (SPSS) data base, furthermore the filled questionnaires were stored in the file, privacy of the questionnaires were maintained throughout the study. The knowledge scores were calculated by assigning a score of “1” to right answers, and a score of “0” to wrong answers and “I don't know.” Similarly, the practice score was calculated. To obtain the overall mean knowledge and practice scores, the total knowledge items and the total practice items were computed, respectively.

A convenient sampling technique was used to collect the data from the students. Data were collected via personally visiting the College of Nursing during workdays. Before the process of data collection, the study objective was explained, and written informed consent was obtained from each student. Students were given adequate time to complete the questionnaire. For the purpose of data collection, a researcher was appointed from the College of Nursing to follow up with respondents. In addition, this study was followed principle of Helsinki ethical for medical research involving human subjects [24].

### 2.3. Sample Size Estimation

The sample size was calculated using an electronic calculator, namely Raosoft, with a confidence interval of 95% and a margin of error of 5% [25]. A sample size of 254 was calculated by referring to the total number of registered students at first year, second year, and third year from the college of nursing records, which was 400 in 2019, but we decided to survey 320 students in an attempt to insure higher reliability.

### 2.4. Statistical Analysis

The collected data were checked for completeness of the questionnaires, where if the participants miss more than two items in the study was considered as a missing and removed from the study. The data were analyzed using the IBM SPSS Statistics 22 (IBM Inc., Chicago, IL, USA) software. Descriptive statistics, frequencies, and percentages were used to summarize the data. Mean knowledge and practice scores were calculated for the knowledge and practice questionnaires. Shapiro–Wilk test, were used to test the normality of distribution, and the data were not normally distributed. The nonparametric tests such as Mann–Whitney *U* test and Kruskal–Wallis test were used, to find any difference between the knowledge and practice scores with respect to demographics characters of the participants. A *p* value of less than 0.05 was considered statistically significant.

## 3. Results

The number of questionnaires returned was 320, of which 16 (5.0%) were excluded from the study due to incomplete responses, leaving a total of 304 valid questionnaires and a response rate of 95%. Of the respondents, majority 201 (66.1%) of them were males and 53% (n = 161) of them were between 21 and 25 years old. The mean age of the students was 21.59 ± 1.93 (CI = 21.3740–21.8102). Most of the students, 129 (42.4%), were second year and were single (284, 93.4%). A detailed description of participant characteristics is presented in Table 1.

Table 2 shows participants' responses to statements in the second part of the questionnaire on hand-washing knowledge. Among the 304 respondents, the majority (94.2%) believed that direct and indirect contacts were the most important routes for the transmission of healthcare-associated infection. Most of the participants (93.2%) agreed that proper and consistent hand washing prevented infections in health facilities. Similarly, 92.1% agreed that effective hand washing consisted of wetting, soaping, applying friction, rinsing, and drying adequately. Most of the participants (89.8%) agreed that using disinfectants during hand washing decreased the bacterial load on hands, and 78% believed that alcohol had a greater ability to eradicate microorganisms compared to water.

Table 3 shows the responses of participants to the statements in part three of the questionnaire, related to hand washing practice. The majority (83.2%) always washed their hands before and after contact with patients, while 89.5% washed their hands after contact with body secretions. A majority (79.6%) washed their hands before performing any clean and aseptic procedure, while 83.6% reported using soap during hand washing. Over half (59.9%) said they used alcohol-based hand rub.

As shown in Table 4, the mean knowledge score for male students was 9.63 ± 1.48, while the score for females was 10.09 ± 1.27. Additionally, the mean practice score for the females was 5.00 ± 1.25, followed by males, at 4.62 ± 1.46. Our results found a significant difference in mean knowledge score with respect to gender (*p*=0.004). Similarly, there was significant difference between mean practice score for hand washing between male and female (*p*=0.037). There was a no significant difference between age group, years of study and marital status of the participants with respect to hand-washing knowledge score (*p*=0.898; *p*=0.130; *p*=0.206), and practice score (*p*=0.135; *p*=0.537; *p*=0.628), respectively.

## 4. Discussion

It has been shown that nursing students involved in professional contact with patients can act as a source for the transmission of infection [19]. Therefore, it was important to educate nursing students and health professionals about the importance of hand washing in their healthcare practice. However, it was a well-known, evidence-based fact that hand washing were an effective method for inhibiting the transmission of infections to patients in healthcare settings [18–21, 26], while good knowledge and practice of hand washing was important in ensuring a healthy environment for both patients and healthcare workers. The use of disinfectants or soap and water in hand washing has been promoted worldwide, including by Saudi Arabia's Ministry of Health and the World Health Organization, for preventing the spread of infectious diseases [26, 27]. The present study had assessed the knowledge and practice of hand washing among nursing students at the King Saud University College of Nursing in Riyadh, Saudi Arabia. This study could contribute to the literature on hand washing and help to raise awareness about the need for hand washing among present and future healthcare professionals.

Our study found that the majority of Saudi nursing students had an adequate knowledge regarding hand hygiene, which was similar to findings in other studies [1, 28]. However, this percentage was higher than that derived in a 2018 study by Jemal, wherein only 60% of health professionals were found to have good hand-washing knowledge [15]. It was also higher than that found by a study conducted in Saudi Arabia by Cruz and Bashtawi, wherein most respondents had moderate (58.6%) knowledge of hand washing [19]. Additionally, the findings from the current study revealed that students might help in controlling the spread of infection through proper education and by creating awareness about the importance of hand washing with their patients, particularly as regards the prevention of droplet infection.

The nursing students demonstrated an awareness of the importance of hand washing and of maintaining an infection-free environment. The fact that 90% acknowledged that hand washing was the single most effective mechanism for preventing the spread of infection was encouraging. These findings were consistent with those in previous studies, wherein most nurses agreed that hand washing was relevant to disease control [14, 15]. In addition to this, the majority agreed that direct or indirect contact was the most important route for the transmission of healthcare-associated infection. A majority also agreed that proper and consistent hand washing prevents infections in health facilities. These results were similar to those found among healthcare professionals by Jemal [14].

Our study found that 84% of nursing students had good hand-washing practice. This was higher than the percentage found in a 2015 study by Cruz and Bashtawi, in which 68.7% of Saudi nursing students had moderate hand washing practice [19]. It was also higher than the result found in a study by Jemal, wherein only 43.96% of healthcare professionals had good hand washing practice [15]. This might be due to the extra-curricular activities in Saudi education, as the Ministry of Health provides multiple health education programs free of charge to healthcare students. In addition to this, knowledge and good practice were associated with the effects of the availability of disinfectants and participants' beliefs on remembering hand washing or always thinking about hand washing after every procedure with a patient. However, several studies reported that the main reasons for practitioners not washing their hands were the side effects of hand washing products, the unavailability of disinfectants, a lack of time, not having sufficient knowledge about hand hygiene, and forgetfulness [11, 29, 30].

Most of the participants in the present study (83.2%) said that they wash their hands before and after contact with patients. This finding was higher than those of previous studies conducted in Ethiopia [1, 15, 31, 32], Nigeria [14, 33], Sri Lanka [34], and Saudi Arabia [19]. Other international studies reported moderate hand washing practice [27, 35] and addressed the importance of hand washing among different populations [22, 27]. Studies have found that hand-washing knowledge and practice were more prevalent in the nursing profession than in any other healthcare profession [36, 37]. Additionally, earlier studies reported that hand washing was more prevalent among nurses than amongst physicians [28, 37]. A 2018 study by Humran and Alahmary carried out among medical students and other healthcare profession students reported that knowledge of hand washing was higher amongst nursing students (84.22 ± 12.98) than amongst other healthcare professionals [26]. A 2019 study by Alotaibi et al. in Saudi Arabia found only moderate (50–75%) levels of knowledge and practice of hand washing among clinical-year medical students [38]. However, a 2018 study by Aledeilah et al. found that 70% of medical students adhered to hand washing practice, compared to a lower percentage of nurses and senior medical staff [35]. Additionally, current research showed that female nursing students performed better than male students in terms of hand washing, these findings were consistent with earlier findings both nationally and internally, who reported females nursing students had good knowledge and practice of hand washing [18, 39]. The greater knowledge amongst female students than amongst other male nurses and other professionals was likely due to the nursing students' high levels of daily contact with patients and the emphasis on this issue in the nursing curriculum. On the other hand, the mindset of the nurse is the next important factor that affects how strictly they adhere to hand hygiene requirements [40]. A female nurse always exhibits a positive attitude toward her job, which may encourage nurses to adhere to the habit of washing their hands [40]. According to a study, female nurses adhere to policies and procedures more frequently than male nurses do [40].

Therefore, it was important to educate nursing students and health professionals about the importance of hand washing in their healthcare practice to raise awareness of the value of hand hygiene among all HCPs, posters and other visual aids demonstrating its significance must be exhibited. Further research could help us to better understand the levels of hand-washing knowledge and practice among different groups and promote awareness of the proper techniques to stop the spread of infection. We recommended repeating the study among other healthcare professions and populations, using a larger sample size.

This study had several limitations. Firstly, it was limited to a single institution. Secondly, it used entry-level junior nursing students who were Arabic speakers; it does not reflect the whole Arabic population and does not represent all university students within the Kingdom. Thirdly, there were no people checking whether students actually washed their hands, so all questionnaire responses are reported, and results may be affected by some bias. Lastly, the data were obtained through self-administration, and thus bias could have been introduced as respondents may have reported higher levels than were actually reflected in their knowledge or practice.

## 5. Conclusions

This study revealed that the majority of Saudi students had good levels of both knowledge and the practice of hand washing. The female participants were found to have better knowledge and practice in comparison to male participants. Moreover, the current findings suggested the need of more efforts to increase the awareness about the importance of hand washing to prevent HCAIS. Furthermore, an extensive health education program on the topic in the nursing curriculum was warranted. We recommended repeating the study among other healthcare professions and populations, using a larger sample size. Further research could help us to better understand the levels of hand-washing knowledge and practice among different groups and promote awareness of the proper techniques to stop the spread of infection.

## Figures and Tables

**Table 1:** Demographic information of study participants (*n* = 304).

Characteristics	Frequency (*n*)	Percentage (%)	Mean age and confidence intervals (CI)
Gender			
Male	201	66.1	
Female	103	33.9	
Age (in years)			
19-20	125	41.1	Mean age (21.59 ± 1.93) 21.3740–21.8102 (CI)
21–25	161	53	
26 and over	18	5.9	
Year of course			
First year	76	25.0	
Second year	129	42.4	
Third year	99	32.6	

**Table 2:** Participants' responses pertaining to knowledge of hand hygiene.

Statements	Yes		No		I don't know	
	*n* ^*∗*^	%	*n*	%	*n*	%
Direct or indirect contact are the most important routes for the transmission of healthcare-associated infection	286	94.2	09	2.9	9	2.9
Proper and consistent hand-washing prevents infections in health facilities	283	93.2	15	4.9	6	1.9
There is no need for hand-washing for those who perform their activity with caution	262	86.2	42	13.8	—	—
There is no need for hand-washing if gloves are properly worn	266	87.5	37	12.2	—	—
Health professionals should always wash their hands immediately when they arrive at health institutions	282	92.8	20	6.6	2	0.7
Hand washing should be practiced routinely even when gloves are worn	284	93.4	18	5.9	02	0.7
Effective hand-washing consists of wetting, soaping, applying friction, rinsing, and drying adequately	280	92.1	23	7.6	01	0.3
Hands should be washed at least for 10–15 seconds	225	74	78	25.7	01	0.3
Using disinfectants during hand-washing decreases bacterial load on hands	273	89.8	30	9.9	01	0.3
Health professionals should wash their hands or use antiseptic hand rub before putting on or after the removal of gloves	283	93.1	20	6.6	—	—
Alcohol has a superior ability to eradicate microorganisms compared to water	237	78.0	66	21.7	—	—
Hand-washing is the single most effective mechanism to prevent the spread of infection	273	89.8	29	9.5	02	0.7

^*∗*^Missing response; *n* = frequency; % = percentile.

**Table 3:** Participants' practice regarding hand hygiene.

Statements	Always^*∗*^		Sometimes		Never	
	Frequency (*n*)	Percentage (%)	Frequency (*n*)	Percentage (%)	Frequency (*n*)	Percentage (%)
Wash hands before and after contact with patients	253	(83.2)	50	(16.4)	0	(0)
Wash hands after contact with body secretions	272	(89.5)	27	(8.9)	0	(0)
Wash hands before performing any clean and aseptic procedures	242	(79.6)	57	(18.8)	0	(0)
Apply soap during hand-washing	254	(83.6)	45	(14.8)	0	(0)
Use alcohol-based hand rub for hand hygiene	182	(59.9)	117	(38.5)	0	(0)
Dry hands after hand-washing	223	(73.4)	76	(25)	0	(0)

^*∗*^Missing response.

**Table 4:** Variance analysis of hand hygiene knowledge and practice scores across demographics.

Characteristics	Knowledge score mean ± SD (Median (IQR))	*p* value	Practice score mean ± SD (Median (IQR))	*p* value
Gender				
Male	9.63 ± 1.48	0.004	4.62 ± 1.46	0.037^*∗*^
	10 (2.0)		5 (2.0)	
Female	10.09 ± 1.27		5.00 ± 1.25	
	10 (1.0)		5 (1.50)	
Age group		0.898		0.135^*∗∗*^
19-20 years	9.78 ± 1.40		4.64 ± 1.41	
	10 (2.0)		5 (2.0)	
21–25 years	9.77 ± 1.45		4.75 ± 1.43	
	10 (2.0)		5 (2.0)	
>26 years	9.94 ± 1.30		5.38 ± 0.84	
	10 (1.25)		6 (1.0)	
Year of study				
First year	10.12 ± 1.18		4.78 ± 1.45	0.537^*∗∗*^
	10 (1.0)		5 (2.0)	
Second year	9.57 ± 1.58	0.130	4.64 ± 1.47	
	10 (2.0)		5 (2.0)	
Third year	9.81 ± 1.32		4.86 ± 1.27	
	10 (2.0)		5 (2.0)	

^*∗*^ = Mann–Whitney *U* test ^*∗∗*^Kruskal–Wallis test.

## Data Availability

The data used to support the findings of this study are available from the corresponding author upon request.

## Abbreviations

HCAI: Healthcare-associated infection.
